# Preterm Birth: A Narrative Review of the Current Evidence on Nutritional and Bioactive Solutions for Risk Reduction

**DOI:** 10.3390/nu11081811

**Published:** 2019-08-06

**Authors:** Tinu M. Samuel, Olga Sakwinska, Kimmo Makinen, Graham C. Burdge, Keith M. Godfrey, Irma Silva-Zolezzi

**Affiliations:** 1 Nestlé Research, 1000 Lausanne, Switzerland; 2 School of Human Development and Health, Faculty of Medicine, University of Southampton, Southampton SO16 6YD, UK; 3 MRC Lifecourse Epidemiology Unit and NIHR Southampton Biomedical Research Centre, University of Southampton & University Hospital Southampton NHS Foundation Trust, Southampton SO16 6YD, UK; 4 Nestlé Research, Singapore 618802, Singapore

**Keywords:** preterm birth, preterm labor, etiology, nutrition, DHA, probiotics

## Abstract

Preterm birth (PTB) (<37 weeks of gestation) is the leading cause of newborn death and a risk factor for short and long-term adverse health outcomes. Most cases are of unknown cause. Although the mechanisms triggering PTB remain unclear, an inappropriate increase in net inflammatory load seems to be key. To date, interventions that reduce the risk of PTB are effective only in specific groups of women, probably due to the heterogeneity of its etiopathogenesis. Use of progesterone is the most effective, but only in singleton pregnancies with history of PTB. Thus, primary prevention is greatly needed and nutritional and bioactive solutions are a promising alternative. Among these, docosahexaenoic acid (DHA) is the most promising to reduce the risk for early PTB. Other potential nutrient interventions include the administration of zinc (possibly limited to populations with low nutritional status or poor zinc status) and vitamin D; additional preliminary evidence exists for vitamin A, calcium, iron, folic acid, combined iron-folate, magnesium, multiple micronutrients, and probiotics. Considering the public health relevance of PTB, promising interventions should be studied in large and well-designed clinical trials. The objective of this review is to describe, summarize, and discuss the existing evidence on nutritional and bioactive solutions for reducing the risk of PTB.

## 1. Introduction

Preterm birth (PTB) is defined as birth at <37 weeks of gestation or at <259 days since the first day of a woman’s last menstruation, and it is broadly classified into extremely preterm (PT) (<28 weeks), very PT (28 to <32 weeks), and moderate PT (32 to <37 completed weeks of gestation). Moderate PTB is further categorized as early PTB (EPTB) and late PTB (LPTB) depending on whether the infant was born <34 weeks or between 34 and <37 weeks of gestation, respectively [1]. PTB is a risk factor for adverse short and long-term health outcomes. Short term, it is the leading cause of neonatal death and the second cause of all under-5 mortality [2]. Long term, it is associated with increased risks of hypertension, cardiovascular and cerebrovascular diseases, type 2 diabetes, chronic kidney disease, asthma and abnormalities in pulmonary function, and neurocognitive disorders [3,4]. In addition, PTB is associated with increased health care costs [5] and socioeconomic disadvantages in adulthood [6].

Estimates based on most recent data from 107 countries suggest that in 2014 an estimated 10.6% of livebirths worldwide (14.84 million) were preterm, with 81.1% occurring in Asia and sub-Saharan Africa. There are also significant disparities in PTB rates between countries (8.7% in Europe vs. 13.55% in North Africa) [7]. PTB rates have been increasing in most countries with reliable data, attributed to factors including but not limited to, better detection, older maternal age, multiple pregnancies from assisted reproductive technologies, and higher rates of underlying conditions such as diabetes and hypertensive disorders [8]. The reported prevalence may be an underestimate due to lack of routine collection, and within and between country comparisons are challenging due to inconsistencies in reporting pregnancy outcomes and utilizing standard definitions of PTB.

A series of maternal factors have been identified that impact the risk of PTB. Of these, some are non-modifiable, such as history of PTB, extremes in maternal age (<19 and >35 years) [9,10,11], multiple pregnancies [12], short cervical length (CL) [13], uterine abnormalities, prior cervical excision [14], dilation/curettage [15], ethnicity and family history [16], and genetic factors [17,18,19,20]. In addition, some are modifiable, such as nutrition, low socioeconomic status, low body mass index (BMI), obesity, poor pregnancy weight gain, smoking, substance abuse, short inter-pregnancy interval, periodontal disease, bacterial vaginosis, late or no prenatal care, untreated antenatal depression, and the use of assisted reproductive technologies [21].

The clinical presentation of PTB is either “spontaneous” (70%) or “indicated”. Spontaneous PTB is characterized by preterm labor (PTL) with cervical dilation or preterm premature rupture of membranes (PPROM), while indicated PTB is initiated by obstetricians due to complications in absence of labor or PPROM [22,23]. Recent evidence indicates that etiologist of spontaneous and indicated LPTB overlap and are often characterized by placental vascular mal-perfusion lesions. However, the etiologies of EPTB differ, with indicated PTB typically characterized by placental vascular mal-perfusion lesions and spontaneous PTB by placental infectious inflammatory lesions [24]. This suggests that the underlying molecular mechanisms associated with EPTB differ to those of LPTB and thus potentially efficacy of preventive interventions may differ between EPTB and LPTB.

**Figure 1:**
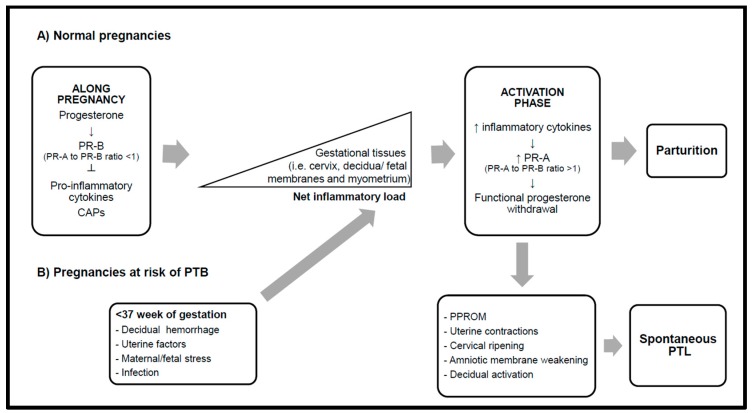
Parturition cascade in normal term and preterm pregnancies. PR-A and PR-B: progesterone receptors A and B, respectively; CAPs: contraction-associated proteins, PTL: preterm labor; PTB: preterm birth; PPROM: preterm premature rupture of membranes.

Differential mechanisms underlying PTB are also inherent to a series of clinical conditions that trigger labor and have been associated with PTB (see Figure 1): (1) decidual hemorrhage caused by placental abruption [25], possibly triggered by infection, inflammation, hypoxia, or oxidative stress [26]; (2) uterine factors such as cervical insufficiency [27] and uterine distension or stretch [21]; (3) maternal mood and distress involving activation of the hypothalamic–pituitary–adrenal axis [28,29,30] and increased production of prostaglandins (e.g., prostaglandin E2) [31,32]; (4) extra and intra-uterine infections [33], as well as intra-amniotic infection (IAI) [34,35], including intrauterine and systemic viral infections [36]; (5) inflammation in the absence of infection (e.g., via production of parturition-triggering cytokines) [37]; and (6) PPROM [38] representing a common final pathway to PTB associated with other of these clinical conditions [39]. 

Although the molecular mechanisms underlying labor onset remain puzzling, it is generally accepted that a “parturition cascade” triggers spontaneous PTL by premature stimulation of pro-inflammatory pathways within the uterus triggered by different clinical conditions (see Figure 1) [33,40,41]. At a molecular level, this cascade is mediated by progesterone and involves the coordinated activation of progesterone receptor-B (PR-B) and its truncated and much less active nuclear PR isoform progesterone receptor-A (PR-A) (see Figure 1) [41]. The balance between inflammation and progesterone activity seems key for timing of delivery.

Novel approaches to reduce the risk of PTB are needed due to the very limited success of different medical strategies to achieve this goal. For example, for women experiencing PTL, tocolytics (cyclooxygenase inhibitors, calcium channel blockers, or betamimetics) are used to stop contractions and delay delivery; unfortunately, these do not remove the underlying stimulus that initiated parturition or reverse parturition [42]. Thus, early identification of risk factors, as well as effective preventive interventions, are needed. Currently, screening, while imperfect, is done based on measuring CL (the strongest clinical predictor of PTB in asymptomatic women), as well as fetal fibronectin levels and CL assessment, the latter in singleton pregnancies with acute PTL symptoms. These approaches remain to be proven in multiple pregnancies [43]. Stopping smoking early in pregnancy has also been shown to reduce the risk of PTB, and other more specific strategies exist for scenarios of low- and high-risk pregnancies [9,44].

In asymptomatic pregnancies with a shortened cervix (CL ≤25 mm) identified at mid-trimester, vaginal progesterone is efficacious for preventing PTB, particularly in multiple pregnancies [45,46]. In singleton at-risk pregnancies and those with history of PTB, vaginal progesterone administered from 16 to 36 weeks of gestation is efficacious [47]. Also, serial CL screening is indicated between 16 and 24 weeks of gestation. In pregnancies with a shortened cervix (CL ≤25 mm) or with history of PTB, vaginal progesterone is the preferred option, with tightening the cervix with a stitch (cerclage) and closing the cervix with a silicone ring (cervical pessary) being alternatives [47,48]. 

Although promising, no approach is uniformly effective as primary-prevention to lower PTB rates because most cases are of unknown cause [49]. Inadequate nutrition preconception and during pregnancy has been associated with the risk of PTB and intervention studies suggest roles for specific nutrients in reducing PTB risk and/or increasing the duration of gestation [50]. The aim of this review is to summarize the evidence on nutritional and bioactive solutions for reducing the risk of PTB.

## 2. Materials and Methods 

Literature search and selection criteria: We conducted a narrative literature review and search using SCOPUS and PubMed databases limited to English language original research, literature reviews and conference abstracts/papers/presentations published before May 2019. For omega-3 fatty acids (with the largest body of evidence today), we reviewed intervention studies of either docosahexaenoic acid (DHA) or eicosapentaenoic acid (EPA) alone or using varying combinations of both, food-based intervention studies, and systematic reviews/meta-analyses. We only reviewed those studies in which long-chain polyunsaturated fatty acid (LC-PUFA) administration was started no later than 12–32 weeks of gestation. The key words used were LC-PUFA, omega 3, eicosapentaenoic acid (EPA) and docosahexaenoic acid (DHA), intervention studies, clinical trials, pregnancy, gestational age at birth, preterm birth, and early preterm birth. For all other macro and micronutrients, we summarized the evidence based on the latest systematic reviews and meta-analyses. The key words used were nutrients, systematic review, meta-analyses, pregnancy, gestational age at birth, preterm birth, and early preterm birth. For probiotics, we included studies that reported PTB as either the primary or secondary outcome, and those that reported outcomes relevant to PTB such as the gestational age at birth or vaginal health during pregnancy; we only included studies where probiotic administration was started no later than 12–32 weeks gestation. 

## 3. Results

### 3.1. The Role of Nutrition in Reducing the Risk of PTB

In the preceding sections, while a broad overview of the pathophysiology of PTB has been presented, there is increasing evidence that infection and/or inflammation are the pathological process for which the molecular pathophysiology has been best defined and a causal link with PTB has been fairly well established [51]. It is therefore of significant relevance to evaluate whether specific diet patterns and nutritional/bioactive interventions targeted to modulate inflammation/infection can be efficacious in reducing the risk of PTB. In the following sections of this review we document the evidence of maternal diet patterns, nutrients, and bioactives on PTB risk reduction and/or increasing gestational age at birth, probably via an anti-inflammatory and/or an immunomodulatory effect.

#### 3.1.1. Evidence from Dietary Pattern Analyses

Observational studies indicate that poor maternal nutrition preconception and during early pregnancy may influence PTB risk [12]. In observational studies, consumption of specific foods such as >1 serving/day of artificially sweetened and sugar-sweetened beverages have been associated with increased PTB risk (adjusted odds ratios: 1.11 and 1.24, respectively), [52], but other studies have found no association [53]. Assessment of dietary patterns found that high scores on a “prudent” dietary pattern (higher intakes of vegetables, salad, onion/leek/garlic, fruit and berries, nuts, vegetable oils, water as beverage, whole grain cereals, poultry, and fiber rich bread, as well as low intake of processed meat products, white bread, and pizza/tacos) was associated with significant reductions in the risk of PTB (by 12%) and spontaneous PTB (by 15%), comparing the highest and lowest thirds of the population. Several mechanisms are postulated through which a prudent diet may reduce PTB risk, including an anti-stressor effect of a low fat diet on the hypothalamic–pituitary–adrenal axis or an anti-inflammatory effect due to an increased antioxidant intake or attributable to a diet low in saturated fat [54]. Adherence to a Mediterranean diet has been linked with a reduced PTB risk. Among Danish women, intake of a Mediterranean diet (fish bi-weekly or more, using olive or rape seed oil, >5 portions of fruit and vegetables/day, meat other than poultry and fish at most twice a week, and at most 2 cups of coffee/day) lowered the risk of EPTB by 72%, although PTB risk was not significantly reduced [55]. Adherence to a dietary pattern similar to Mediterranean diet was associated with a 30% decreased risk of PTD specifically in overweight and obese pregnant women in a French Caribbean island where the population is largely of African descent [56]. Similarly, in a randomized controlled trial (RCT) in healthy Norwegian pregnant women, those adhering to a dietary pattern resembling the Mediterranean diet had a 90% reduction in PTB risk [57]. However, Norwegian women who met the Mediterranean Diet criteria (fish ≥2 times a week, fruit and vegetables ≥5 times/day, use of olive/canola oil, red meat intake ≤2 times/week, and ≤2 cups of coffee/day) did not have reduced risk of PTB [58]. In addition, a vegetarian diet or pre-dominantly plant-based diet, both low in vitamin B12, vitamin D, zinc [59], EPA and DHA [60], as well as marginal intakes or low status of these nutrients have been associated with increased PTB risk [61]. These observational studies are reinforced by three recent systematic reviews showing that a “healthy” dietary pattern during pregnancy higher in fruits, vegetables, legumes and whole grains is associated with a lower risk of PTB (21% to 25% reduced risk) [62,63,64].

#### 3.1.2. Nutrient-Based Interventions

##### Omega 3 Fatty Acids

Omega-3 fatty acids are long-chain, polyunsaturated fatty acids (PUFAs) of plant and marine origin. Alpha linolenic acid (ALA) and linoleic acid (LA) cannot be synthesized by the human body and must be derived from dietary sources. ALA is the parent omega-3 fatty acid that can be converted into longer chain n-3 PUFA including EPA and DHA [65]. The conversion of ALA to EPA and DHA is inefficient in humans and varies markedly between individuals; only 8–12% ALA is converted to EPA and less than 0.05% to DHA [66]. The efficiency of the conversion is dependent on epigenetic and genetic processes influencing the transcription of the fatty acid desaturase (*FADS*) genes [67]. Dietary factors can reduce conversion efficiency. In particular, greater conversion is observed among women compared to men, probably due to the influence of estrogen or other hormones [65,66]. It is recommended to obtain EPA and DHA preformed from additional dietary sources including fish/seafood and oils from marine animals, such as fish oil and cod liver oil. DHA intake across the world is variable [68,69]. 

The following sections briefly describe the evidence relating to EPA and DHA and PTB risk reduction. The first evidence that omega 3 fatty acids may play a role in reducing the risk of PTB was observed in women from Faeroe Islands who consumed a diet high in fish and showed an increased duration of gestation (4 days) and birth weight (194 g) compared to Danish women [70]. Additional supportive observational evidence included a prospective study in pregnant women reporting that non-consumers of fish had a shorter gestational length and higher odds of PTB, compared to fish eaters [71], another showing that any intake of seafood was associated with a lower prevalence of PTB [72], and a dose–response relationship observed for the association between seafood intake and risk of PTB [73]. However, other large cohort studies in the United States [74] and the United Kingdom [75] did not support the association of n-3 PUFA intake and/or seafood intake with length of gestation or PTB risk. 

Ten clinical trials have investigated the role of omega 3 fatty acids on the duration of gestation and/or the incidence of PTB and/or EPTB [76,77,78,79,80,81,82,83,84,85] (Table 1). Eight of these 10 trials were interventions constituting of either DHA or EPA alone or used varying combinations of both, and two were predominantly food based [84,85]. Only two trials were carried out among pregnant women from low or middle-income countries (Mexico and Chile) [78,84]. Three trials were conducted in women with high-risk pregnancies (history of PTB, history or high risk of developing intra-uterine growth restriction (IUGR), or pregnancy-induced hypertension (PIH), women diagnosed with pre-eclampsia in current pregnancy or twin pregnancies) [80,81,82]. While most of the trials started the intervention around mid-pregnancy and followed through to delivery, one trial recruited women as early as 12–14 weeks gestation [82], and another at approximately 30 weeks of gestation [83].

The Kansas DHA Outcome Study (KUDOS trial), demonstrated that a daily administration of 600 mg DHA/day in pregnant women improved gestation duration (2.9 days) [76], and while an overall reduction in PTB was not observed, there was a reduction in EPTB (<34 weeks gestation). The results of this study are in accordance with those of another very large Australian trial (DHA to Optimize Mother Infant Outcome, DOMInO) in which positive findings in secondary outcomes such as an increase in the duration of gestation (1 day) and a 51% reduced risk of EPTB were also observed [77]. A trial among Danish pregnant women also demonstrated an improvement in the duration of gestation (4 days) with fish oil supplementation (2.7 g n-3 fatty acids) compared to olive oil. In this study, the effect of supplementation on the length of gestation seemed to depend on the habitual intake of fish. Among women who had the highest intake of fish at randomization, no difference could be detected between the groups, while in the women with the lowest intake for fish; a significant difference of 7.4 day was observed [83]. In contrast, a large trial among predominantly middle-class Mexican women from urban areas with access to health care indicated no benefit of supplementation of 400 mg DHA/day on gestational age, incidence of PTB, birth weight, length, or head circumference [78]. The results from the Norway [79], United Kingdom [81] and the Netherlands [82] trials concurred with the Mexican trial and did not find that omega 3 fatty acids lengthened the duration of gestation or reduced the incidence of PTB or early PTB. Two food-based interventions have assessed the effect of a dairy product fortified with multiple micronutrients, ALA and LA [84] or DHA-enriched eggs [85], on the length of gestation and birth weight. Both these trials reported a lower incidence of EPTB and an increase in the length of gestation. The latter trial [85] demonstrated an increase of 6 days of gestation with as little as 133 mg DHA/day.

Many factors may influence the response to supplementation and variability in outcomes observed in these trials, including variability in the dose of n−3 LC-PUFA, differences in the source of DHA, population differences in gestation duration, PTB rates and birth weight within the control arm, differences in habitual intakes of DHA from food, differences in baseline n-3 fatty acid status, and high-risk pregnancies with a history of PTB. The role of *FADS* gene variants as determinants of PUFA levels should also be considered as depending on genetic variants, requirements of dietary PUFA or LC-PUFA intakes to achieve comparable biological effects may differ [86] In the Child, parent and health: lifestyle and genetic constitution (KOALA—acronym of the Dutch title) birth cohort, both maternal DHA intake and the maternal FADS rs174556 Single Nucleotide Polymorphism (SNP) genotype were associated with pregnancy duration, and women who were homozygous for the minor allele (indicating their lower n–3 LC-PUFA interconversion and hence higher dependence on dietary supply) had significantly shorter pregnancies (2 days) [87]. In all of the trials with DHA supplementation, PTB or EPTB was never a primary outcome, but rather a secondary or safety-related outcome. Two ongoing RCTs (Assessment of DHA On Reducing Early preterm birth, ADORE and Omega-3 fats to Reduce the Incidence of Prematurity, ORIP) will examine the efficacy and safety of high dose DHA supplementation to reduce early PTB as a primary outcome (1000 mg/day and 800 mg/day DHA, respectively) [88,89].

Six meta-analyses/systematic reviews/Cochrane reviews have evaluated the effect of EPA + DHA (n-3 LC-PUFA) supplementation during pregnancy on gestation duration and risk of PTB and found that supplementing pregnant women with n−3 LC-PUFA appears to be beneficial in reducing the risk of EPTB (magnitude of the effect ranging from 26% to 61%) [90,91,92,93,94]. However, the clinical relevance of a minor increment in gestation duration is questionable. The most recent Cochrane systematic review of RCTs comparing omega-3 fatty acids during pregnancy with placebo or no omega-3 showed a risk reduction of 42% for EPTB (nine RCTs, 5204 participants; high-quality evidence) and 11% for PTB (26 RCTs, 10,304 participants; high-quality evidence). The mean gestational length was also greater in women who received omega-3 LC-PUFA (mean difference (MD) 1.67 days, 41 trials, 12,517 participants; moderate-quality evidence) [95]. Another recent meta-analysis (nine RCTs) demonstrated that n-3 LC-PUFA are effective at reducing the risk of EPTB by 58%, any PTB by 17%, increasing the length of gestation by 1.95 weeks and increasing birth weight by 122.1 g, and that these effects did not differ according to the risk status of women or dose or timing of the intervention [93]. Evidence from recent trials that have used DHA alone (600 mg DHA/day) [76] or DHA as the main n-3 fatty acid in terms of dose (800 mg DHA/day + 100 mg EPA/day) [77] also showed reduction of the risk of EPTB (magnitude of the effect from 51.6% to 87.5%). It also appears that higher doses of DHA (≥600 mg DHA/day) may be needed to have a protective effect, as trials providing <600 mg DHA/day have not found a reduction in EPTB, and ongoing studies will test the efficacy of doses up to 1000 mg DHA/day. 

It could be hypothesized that DHA supplementation reduces the inflammation responsible for both cervical ripening and spontaneous EPTB [87] or that it increases circulating EPA through enhanced biosynthesis via retro conversion from supplemented DHA [96]. Other mechanisms may explain a lengthened duration of gestation. EPA competes with arachidonic acid (ARA), which is the source of pro-constriction mediators such as the 2-series prostaglandins E2 and F2α that can cause contraction of myometrium and cervical ripening and result in an increase the production of prostacyclins (PGI2 and PGI3), with a relaxant effect on the myometrium [70]. Omega 3 fatty acids are also thought to have an ‘‘antiarrhythmic’’ effect on the myometrium that may delay the initiation of labor [97]. Increased intake of marine PUFA is hypothesized to attenuate inflammation by modifying the membrane phospholipid fatty acid composition, altering the physical properties of the cell membrane such as membrane fluidity, through its effects on cell signaling pathways or by altering the pattern of the lipid mediators produced [98].

##### Other Macro and Micronutrients

Zinc is key for protein synthesis, cellular division, and nucleic acid metabolism [99]. Inadequate intakes (diets lacking in animal food sources rich in zinc) coupled with the limited zinc absorption (high consumption of phytate from cereals) and chronic infections result in reduced maternal plasma concentrations, resulting in reduced supply of zinc to the fetus [100,101]. Zinc deficiency alters the circulating levels of a number of hormones associated with the onset of labor such as progesterone and prolactin [100]. Zinc supplementation has been proposed to reduce the incidence or the severity of maternal infections, and thereby lower the risk of PTB [102]. A Cochrane review (16 RCTs, 7637 women) demonstrated moderate quality evidence of a small but significant 14% reduction in PTB with antenatal supplementation of zinc alone or in combination with other micronutrients compared to placebo. However, this was not accompanied by a similar reduction in the proportion of low birth weight infants or a difference in the gestational age at birth. Most of the studies included women from low- and middle-income settings who had, or were likely to have, low zinc concentrations and overall low nutritional status, making these findings particularly relevant in low-income countries with high perinatal mortality [103]. To the contrary, a more recent systematic review found no association between maternal zinc status and spontaneous PTB, however the authors expressed uncertainty on the evidence due to the heterogeneity in the studies included and the need for further studies populations at increased risk of zinc deficiency [104].

The hormonal form of vitamin D3 (1α,25-dihydroxyvitamin D3) plays a role in the mineralization of the skeleton and regulation of parathyroid hormone, and affects physiological pathways involved in PTB, including inflammation, immunomodulation, and transcription of genes involved in placental function [105]. Vitamin D deficiency in reproductive-age women is widespread and low maternal vitamin D status during pregnancy is a risk factor for various adverse birth outcomes including PTB [106]. Vitamin D is suggested to have an effect on PTB due to its immunomodulatory role and anti-inflammatory effects [107,108]. Two recent meta-analyses of observational studies have shown that vitamin D deficiency as indicated by serum 25 hydroxyvitamin D (25-OHD) levels <50 nmol/L is associated with an increased risk of PTB (by an odds of 1.25 to 1.29 times) [109,110]. Another meta-analysis of observational studies found that 25(OH) D levels >50 nmol/L was associated with longer gestation duration (difference of 0.2 week) [111]. A Cochrane review showed that supplementation with vitamin D alone versus no intervention/placebo reduced the risk of PTB by 64% (three trials and 477 women, moderate quality evidence), while supplementation with vitamin D and calcium versus no treatment/placebo significantly increased the risk of PTB (3 trials and 798 women); however, most trials included were of low methodological quality [112] Recent high quality trials such as The Maternal Vitamin D Osteoporosis Study (MAVIDOS) [113] were not included in the review; in the MAVIDOS trial PTB was reported only as a safety-related outcome but there was no effect of vitamin D supplementation on PTB.

Magnesium is key in the regulation of body temperature, synthesis of nucleic acids and proteins, and maintenance of electrical potentials in nerves and muscle membranes. Ionized and total magnesium levels are shown to decrease with increasing gestational age [114]. Insufficient magnesium intake is common in women [115] and magnesium deficiency during pregnancy is associated with a higher risk of chronic hypertension, preeclampsia, placental dysfunction and premature labor [116]. Reduced placental vascular flow is considered responsible for placental insufficiency and fetal intra-uterus growth restriction and magnesium is believed to have an immediate effect on placental vascular flow [117]. A 2001 Cochrane review (seven trials, 2689 women) reported that oral magnesium supplementation starting before 25 weeks of gestation compared to placebo was associated with a 27% reduction in risk of PTB, without any effect on gestational age at birth. However, when one of the studies that had a cluster design was excluded, there was no effect of magnesium supplementation on any of the outcomes [118]. A recent update of this review reported no significant differences in outcomes such as gestational age at birth (five trials, 5564 women) and PTB (seven trials, 5981 women) between the magnesium supplemented group and the control group [119]. Overall, both reviews reported a lack of high quality evidence. Magnesium sulphate has been used as a tocolytic agent to inhibit uterine activity in women in the setting of preterm labor with the aim of preventing PTB. However, a Cochrane review (37 trials, 3571 women), concluded that magnesium sulphate is ineffective at delaying birth or preventing PTB, has no clear benefits on neonatal and maternal outcomes and may be associated with an increased risk of fetal, neonatal, or infant mortality [120]. A multicenter double-blind, placebo-controlled randomized clinical trial of oral magnesium citrate supplementation (the BRAzil MAGnesium (BRAMAG) trial) among high-risk pregnant women starting at 12 to 20 weeks of gestation through to delivery is currently ongoing [121], with the primary perinatal outcome being a composite of PTB < 37 weeks gestation, stillbirth >20 weeks gestation, neonatal death < 28 days, or Small for Gestational Age (SGA) birthweight <3rd percentile. 

Calcium plays a role in nerve cell function, muscle contraction, enzyme and hormone actions, and bone mineralization. A recent Cochrane review suggested that there are no benefits of calcium supplementation during pregnancy in reducing the risk for either PTB or EPTB. The significant heterogeneity among studies (13 trials), led the investigators to perform sub-group analyses stratified by the total dose of calcium per day (<1000 mg/day or ≥1000 mg/day), starting time of calcium supplementation (before or after 20 weeks), and type of calcium (calcium carbonate, lactate and gluconate). There were no statistically significant differences between sub-groups for either the starting time of supplementation or the type of calcium [122]. The effects of baseline calcium intake (*n* = 5 trials) and risk for hypertensive disorders of pregnancy (*n* = 4 trials) have also been studied, however, no protective effect of calcium supplementation on PTB risk reduction was observed [123]. Iron is key for oxygen transport from lungs to tissues, in energy transfer and facilitating oxygen use and storage in muscles. Iron deficiency is the most common nutrient deficiency among pregnant women and results from an increased requirement for iron during pregnancy, a diet poor in absorbable iron, and parasitic infections [124]. Folate is essential for the synthesis of nucleic acid, amino acids, phospholipids and, consequently, lipoproteins, cell division, tissue growth, and DNA methylation. Supplementation with folic acid in the immediate period before and early in pregnancy reduces the risk of neural tube defects [125]. Observational studies show that both anemia [126] and iron deficiency [127] are associated with increased risk of PTB. Recent Cochrane reviews have analyzed the efficacy of a range of interventions containing iron alone, folic acid alone or iron and folic acid together on reduction of PTB. There were no reported differences in the number of women experiencing PTB receiving supplements with iron alone versus no treatment/placebo (six trials, 1713 women) [124], folic acid alone (three trials, 2959 women) [128], daily iron and folic acid supplements versus no treatment or placebo (three trials, 1497 women), or any supplements containing iron and folic acid versus same supplements without iron nor folic acid or placebo (three trials, 1497 women) [124,128]. The studies were of low quality and used a heterogeneous definition of PTB ranging from anywhere between 36 to 38 weeks.

Vitamin A plays a role in visual function and modulation of the expression of genes involved in immune function, reproduction, tissue growth and embryonic development. Vitamin A deficiency (low serum and breast milk vitamin A concentrations) is highly prevalent among pregnant women from Asia, South Asia, and Africa [129]. Vitamin A or beta carotene supplementation during pregnancy has been shown to improve hematologic status of women by improving hemoglobin levels and reducing the risk of anemia [130]. A large trial in Nepal demonstrated that vitamin A supplementation during pregnancy was associated with a 40% reduced risk for pregnancy-related maternal mortality [131]. However, a meta-analysis reported no significant effect of vitamin A supplementation either on PTB (five studies) or EPTB (two studies) risk reduction, and only one trial among South African women reported a significant 33% reduction in the prevalence of PTB and a 66% reduction in EPTB, but this effect disappeared after excluding multiple pregnancies [130].

Sub-optimal micronutrient intakes and micronutrient deficiencies during pregnancy are a global problem and have been associated with placental oxidative stress and complications of pregnancy such as PTB and preeclampsia [100,132]. Although mechanisms linking the use of multivitamins with PTB are not fully understood, they are thought to be involved in the process of normal placentation, and deficiencies in vitamin B12 and folate have been implicated in the development of defects within the placental vascular bed [133]. Impaired placentation has been associated with recurrent PTB [134]. Results from the Danish National Birth Cohort show that peri-conceptional multivitamin use was associated with a 16% reduced risk of PTBs, with a 20% risk reduction of preterm labor in non-overweight women [135]. However, a recent Cochrane review showed no significant differences in PTB between women who were supplemented with Multiple Micronutrient (MMN) containing iron and folic acid versus those receiving iron, with or without folic acid (15 trials, 90892 women, high quality evidence). A sub-group analysis, however, showed that MMN supplementation led to significantly fewer PTB in women with a BMI <20 kg/m² (RR 0.85, 95% CI 0.80–0.90, *n* = 4) [136]. 

Balanced protein-energy supplementation (protein <25% of the total energy content) was found to reduce the risk of intrauterine growth retardation (IUGR) (23% risk reduction) [137] and was associated with modest increases in maternal weight gain (on average 21 g/week), birth weight (on average 38 g) and a substantial 32% reduction in risk of small-for-gestational-age (SGA) [137]. However, evidence from five trials with 2436 pregnant women reported a non-significant reduction in PTB with balanced protein energy supplementation (without any effect on mean gestational age), possibly related to lack of data on gestational age or problems in gestational age measurement, highlighting the need for confirmatory trials in this area [138].

### 3.2. Role of Probiotics in Reducing the Risk for PTB

The main rationale for intervention with probiotics stems from observational data suggesting a link between vaginal infections, dysbiosis, and PTB [139]. It is universally accepted that a certain proportion of PTB is caused by ascending infections from vagina underlying the importance of vaginal health. Moreover, it has been suggested that vaginal dysbiosis (bacterial vaginosis, BV) could trigger an inflammatory cascade leading to PTB even in the absence of ascending infection. Antibiotics such as metronidazole are the standard of care treatment for BV, but there is conflicting evidence whether such treatment results in the risk reduction of PTB [140]. The modest, at best, efficacy of antibiotics is not be surprising, because bacterial vaginosis is characterized by the absence of lactobacilli in addition to the presence of specific pathogenic organisms, and antibiotics cannot restore the depleted lactobacilli. *Lactobacillus* probiotics could fulfill this role through the production of lactic acid, lowering vaginal pH and helping to prevent the growth of potentially pathogenic microorganisms through production of hydrogen peroxide, bacteriocins, and surface-binding proteins that inhibit adhesion of pathogens. Indeed, oral or vaginal administration of probiotics have improved vaginal microbiota composition or alleviated BV in several studies [141]. In addition, and independently of maintenance of vaginal health, oral probiotics may act directly in the gut, down-modulating local and systemic inflammation [142].

#### 3.2.1. Probiotic Intervention studies

We identified 71 publications derived from 21 individual clinical studies that described health outcomes following the administration of probiotics during pregnancy. One reported PTB as the primary outcome and four as a secondary outcome. The rest reported other outcomes informative of PTB such as the gestational age at birth or vaginal health during pregnancy. Of the 21 studies, only eight, where probiotic administration was started no later than at 12–32 weeks of gestation, were considered here (Table 2).

#### 3.2.2. The effect of Oral Probiotics on PTB Rates

Two studies (NCT00217308 and NCT00303082), were designed to test the effect of oral administration of *Lactobacillus rhamnosus* GR-1 and *Lactobacillus reuteri* RC-14 or placebo on the incidence of BV and PTB (co-primary outcomes [143]). Both were discontinued due to difficulties with recruitment, but the partial results were published [144]. The PTB rate was 1.6% in the treatment and 3.3% in the placebo group (RR:0.495; 95% CI: 0.17,1.43; *p* = 0.14). The overall PTB rate (2.5%) was lower than the national average for Brazil (9%) [8].

The same combination of probiotics was tested in a German study [145] with BV as the primary outcome, and PTB rate reported as a secondary outcome. The investigators expected BV rates of 28–42%, but only 6% at baseline was observed, decreasing to 2.5% by the end of the study. There were no differences in BV incidence between the probiotic and placebo groups. PTB rate (4%) was lower than the national average of 9.2% [8]. 

PTB rates were reported as a secondary outcome in two studies testing different combinations of probiotics. *Lactobacillus rhamnosus* GG and *Bifidobacterium lactis* Bb12 were tested in Finland [146], and *Lactobacillus rhamnosus* LPR and *Bifidobacterium lactis* NCC 2818 in the Philippines [147]. Both studies found PTB rates much lower than national averages reported by WHO, 1.7% vs. 5.5% in Finland and 2.4% vs. 15% in the Philippines [8].

In all the above studies, no firm conclusions could be drawn regarding the effect of probiotics on PTB due to its lower than expected rates, but also raised questions about causes of the observed low PTB rates. The enrollment biases resulting in inadvertent exclusion of women at highest risk is the likely explanation.

Four studies in which probiotic administration was started at 24–32 weeks of pregnancy reported that the gestational age and its range did not differ between the probiotic and the placebo groups [148]. However, because direct data on PTB rates were not reported and the statistical power of the studies with regards to PTB is unknown, no conclusions on the potential effect of probiotics on PTB can be drawn from them. 

A prospective cohort study conducted based on Norwegian Mother and Child Cohort Study (MoBa) examined the consumption of probiotic milk during pregnancy and concluded that the intake in early pregnancy (around 17 weeks of gestation) was associated with reduced risk of preterm birth, while late intake (around 30 weeks of gestation) was not [149,150]. The main probiotic milks available in Norway at that time contained *Lactobacillus acidophilus* LA-5, *B. lactis* Bb12, and *L. rhamnosus* GG. Regarding systematic reviews and meta-analyses, a 2007 Cochrane review on the efficacy of probiotics for preventing preterm labor [151] concluded that there were insufficient data as only one study with PTB as an outcome (not primary) was found [146]. The review was updated in 2010 with unchanged conclusions. A recent systematic review and meta-analysis which included unpublished data from previous studies concluded that there was no evidence that taking probiotics or prebiotics in pregnancy decreases the risk of preterm birth [152]. However, even though thirteen studies with 2484 participants were included, the overall PTB rate was only 3.6%. Moreover, the probiotic administration was started in early pregnancy (before 18 weeks of gestation) in only two studies. A recent Cochrane review examined the impact of probiotics on prevention of morbidity and mortality in preterm infants, and the rate of PTB [153]. The overall conclusion was that there is insufficient evidence and more research is needed. Therefore, it appears that the question about potential benefits of probiotic administration in early pregnancy remains open.

#### 3.2.3. The Effect of Vaginal Probiotics on Outcomes Related to PTB

Stojanovic et al. 2012 [154] reported a study testing the effect of a vaginal tablet containing *Lactobacillus rhamnosus* BMX 54 on vaginal health. However, the probiotic had a favorable impact on parameters considered indicative of increased risk of PTB or miscarriage, such as the length, consistency and dilatation of the cervix, the level of the presenting part of the fetus as well as the prevalence of pathogenic microorganisms in vaginal and/or cervical swabs, lower vaginal pH, and lower vaginal discharge “whiff test” positivity. Since the study did not follow the women until delivery, and no data on PTB or other birth outcomes are available but the improvement in parameters associated with PTB was observed. Two ongoing studies in US (NCT00635622, NCT02766023), conducted in non-pregnant women, are testing the benefits of vaginal application of *Lactobacillus crispatus* CTV-05 on BV, as recent data indicate that absence of *L. crispatus*, as opposed to any lactobacilli, is most strongly correlated with adverse vaginal health outcomes [155,156]. 

#### 3.2.4. Ongoing Probiotic Studies

In an ongoing study (NCT02693041), *L. rhamnosus* GG or placebo is administered orally in pregnancy starting at 17 weeks gestation, to test the hypothesis that the probiotic decreases the rate of PTB and the incidence of pre-eclampsia (PE) by affecting the inflammatory state. In addition, several registered trials (NCT02430246, NCT02692820, NCT02150655) target the treatment of bacterial vaginosis in pregnancy, all with the combination of *L rhamnosus* GR-1 and *L. reuteri* RC-14. Even though PTB prevention is not a primary outcome in these trials, one of them will report it as a secondary outcome. All trials also refer, in their scientific rationale, to PTB prevention, which could be targeted in subsequent trials once the efficacy of probiotics to decrease BV during pregnancy is proven. 

## 4. Conclusions

Worldwide about 15 million preterm babies are born annually, and despite intensive research the specific mechanisms triggering the PTB remain unclear. An increase in the net pro-inflammatory load has been proposed as main driver of progesterone withdrawal, leading to the onset of parturition. Progesterone has been shown to be the most effective pharmacological intervention to reduce the risk of PTB in singleton pregnancies among at-risk women with a previous PTB, but most approaches tested, mainly directed at at-risk pregnancies, have not proven effective at lowering the rate of PTB, probably because the majority of cases are of unknown cause. Though great effort has been placed on early diagnosis, only a small proportion of PTB are successfully predicted using CL measurements, alone and in combination with fetal fibronectin quantification. In the absence of predictive tests that are sensitive, specific, and feasible to implement, more general approaches for primary prevention are needed. In this respect, nutritional and bioactive interventions seem a promising alternative. This review provides a comprehensive overview of the existing literature on the role of nutritional approaches to reducing the risk of PTB. The beneficial effect of n−3 LC-PUFAs (combinations of EPA and DHA) in reducing the risk of EPTB has been demonstrated in large intervention studies and several meta-analyses. Also, the role of only DHA is supported by two large RCTs. Higher doses of DHA (doses ≥600 mg DHA/day) may be needed to have a protective effect, although the optimal dosage is yet to be determined. The evidence of n−3 LC PUFA and in particular DHA appears to be quite substantial, and the two large ongoing studies should provide further clarity and confirmation as to whether DHA could be brought into clinical practice and recommended for all pregnant women or specific populations at risk. Other nutrients that may help reduce the risk of PTB include zinc (effects might be limited to populations with low overall nutritional status or poor zinc status) and vitamin D (Table 3). The emerging evidence is promising; however, larger and well-designed studies with EPTB and/or PTB as primary outcome are needed before conclusions can be drawn or recommendations made. Current data do not permit any conclusions to be drawn for the efficacy of vitamin A, calcium, iron, folic acid, iron folate, MMN, and probiotics in reducing the risk of PTB. Ongoing studies will elucidate the role of magnesium supplementation and probiotics on reducing risk for PTB. Large-scale clinical trials of promising interventions are needed to provide sound evidence-based recommendations for clinical practice. Due to the heterogeneity in the etiology of PTB, we hypothesize that differential responses to treatment will be identified. Thus, it will be important to include a sufficiently wide and deep selection of high-throughput and comprehensive analyses of the host (e.g., genomics, metabolomics) as well as gut and vaginal microbiome (metagenomics, metabolomics) to allow for the identification of subpopulations and individual responses. If successful, it will be of utmost importance to develop implementation of targeted strategies that enable both practical and affordable scaling-up to cover gaps as well as evidence-based precision nutrition in antenatal care. This is central considering that PTB is a public health problem in both high- and low-income countries.

## Figures and Tables

**Table 1:** Characteristics and efficacy of clinical trials investigating the role of omega-3 fatty acids in reducing risk of early and any PTB.

Ref.	Study				Ingredient, Daily Dosage	Main Results	Comments
	Objective	Design	Population/Sample Size	Duration			
Carlson et al. 2013 [76]	To assess if DHA supplementation can increase maternal and newborn DHA status, gestation duration, birth weight, and length	RCT, DB, PC.	Healthy pregnant women between 8 and 20 weeks of gestation from the USA, *n* = 350	<20 weeks of gestation until delivery	Intervention: 3 capsules/day of a marine algae-oil source of DHA (600 mg DHA/day) Placebo: 3 capsules containing half soybean and half corn oil	Compared to placebo, DHA supplementation resulted in: (1) Longer gestation duration (2.9 day; *p* = 0.041). (2) Fewer infants born <34 weeks of gestation (*p* = 0.025). EPTB reduced by 87.5%. (3) Shorter hospital stays for PT infants (40.8 compared with 8.9 day; *p* = 0.026). (4) Similar PTB incidence between groups, with more EPTB in the placebo group (4.8% vs. 0.6%, *p* = 0.025). (5) Greater birth weight (172 g; *p* = 0.004), length (0.7 cm; *p* = 0.022), and head circumference (0.5 cm; *p* = 0.012). (6) Higher maternal and cord Red Blood Cell-phospholipid-DHA (2.6%; *p* < 0.001).	Women taking supplements <300 mg DHA/day were not excluded. Dietary n−3 LC-PUFA intakes were not assessed Many secondary variables were studied but without adjustment for multiple comparisons. Incidence of PTB and EPTB were secondary outcomes.
Makrides et al. 2010 [77]	To assess if DHA supplementation during the last half of pregnancy has a beneficial effect on maternal depressive symptoms and child neurodevelopment	RCT, DB, PC.	Healthy pregnant women <21 weeks gestation from Australia *n* = 2399	<21 weeks of gestation until delivery	Intervention: 3 capsules/day of DHA-rich fish oil concentrate (800 mg DHA/day A + 100 mg EPA/day) Placebo: 3 capsules/day of vegetable oil containing a blend of rapeseed, Sunflower, and palm oil	Compared to placebo, DHA+EPA supplementation resulted in (1) No differences in the rate of women with depressive symptoms, as well as the cognitive and language composite scores of their children. (2) A small to modest increase in the duration of gestation (precise estimate of effect size could not be determined due to obstetric interventions). (3) Fewer infants born <34 weeks gestation (1.09 % vs. 2.25% adjusted Relative Risk (RR), 0.49; *p* = 0.03), and association with fewer low birth weight infants and fewer admissions to neonatal intensive care. EPTB was reduced by 51.6%.	Dietary intake of n-3 LC-PUFAs was not assessed. The study failed to demonstrate an improvement in primary outcomes such as reduction in depressive symptoms among women and improvement in cognitive and language scores of their children.
Ramakrishnan et al. 2010 [78]	To assess if prenatal DHA supplementation increases gestational age and birth size	RCT, DB, PC.	Healthy pregnant women from 18 to 22 weeks of gestation from Mexico *n* = 1094	From 18 to 22 weeks of gestation until delivery	Intervention: 2 capsules/day of 200 mg of DHA derived from an algal source (400 mg DHA/day) Placebo: 2 capsules/day containing olive oil	Compared to placebo, DHA supplementation resulted in (1) No differences in mean gestational age, PTB, weight, length and head circumference at birth.	
Helland et al. 2001 [79]	To evaluate the effect of n-3 or n-6 long-chain PUFAs on birth weight, gestational length, and infant development	RCT, DB, PC.	Healthy, nulli- or primiparous women in weeks 17 to 19 of pregnancy from Norway *n* = 590	17 to 19 weeks of gestation until 3 months after delivery	Intervention: 10 mL/day of cod liver oil, providing around 2 g daily of the long chain omega-3 fatty acids. Placebo: 10 mL/day of corn oil, providing around 5 g daily of omega-6 fatty acid linoleic acid.	In comparison with placebo, cod liver oil supplementation resulted in (1) No differences in gestational length or birth weight, length or head circumference. (2) Higher concentrations of n-3 fatty acids EPA, DHA, and DHA in umbilical plasma phospholipids. (3) Neonates with high concentration of DHA in umbilical plasma phospholipids (upper quartile) had longer gestational length than those with low concentration (lower quartile; 282.5 (8.5) vs. 275.4 (9.3) days).	Substantial numbers of women excluded from the two groups post randomization due to withdrawals. It does not mention gestational lengths to facilitate undertaking of an ITT analyses. In this population, baseline intake of long-chain n-3 fatty acids was estimated to be relatively high (0.5 g/day) and less than one 1% had a PTB.
Olsen et al. 2000 [80]	To test the preventive effects of dietary n-3 fatty acids on Pre-term delivery, Intrauterine growth retardation, and pregnancy-induced hypertension	Multicenter RCT, PC (4 prophylactic + 2 therapeutic trials)	High risk pregnancies from 19 hospitals in 7 different countries in Europe Four prophylactic trials: previous pre-term (*n* = 232), IUGR (*n* = 280), PIH (*n* = 386) and twin pregnancies (*n* = 579) Two therapeutic trials: threatening pre-eclampsia (*n* = 79) and suspected IUGR (*n* = 63)	From ~20 weeks (prophylactic trials) or 33 weeks (therapeutic trials) until delivery.	Intervention: prophylactic trials (4 capsules/day of fish oil, 1.3 g EPA and 0.9 g DHA) and therapeutic trials (9 capsules/day of fish oil, 2.9 g EPA and 2.1g DHA) (32% EPA, 23% DHA, 2 mg tocopherol/mL) Placebo: identical looking capsules of olive oil (72% oleic acid, 12% linoleic acid)	Compared to placebo, fish oil supplementation resulted in the following among women with a previous Pre-term delivery in the prophylactic trial: (1) Reduced recurrence risk of PTB from 33% to 21% (Odds Ratio (OR) 0.54 (95% Confidence Interval (CI) 0.30–0.98)) (2) Reduced recurrence risk of EPTB from 13.3% to 4.6% (OR 0.32 (95% CI 0.11–0.89)). (3) Longer mean gestational length by 8.5 day (95% CI 1.9–15.2. (4) No effect on PTB in twin pregnancies.	
Onwude et al. 1995 [81]	To determine whether n-3 fatty acid (EPA/DHA) prophylaxis is beneficial in high-risk pregnancies	RCT, DB, PC.	Pregnant women at high risk of developing PIH and asymmetrical IUGR from an antenatal clinic from UK *n* = 233	From around 25 weeks of gestation until 38 weeks of gestation	Intervention: 9 capsules/day of fish oil providing 2.7 g omega-3 fatty acids/day (1.62 g of EPA and 1.08 g of DHA) Placebo: matching air-filled capsules	Compared to placebo, fish oil supplementation resulted in (1) No difference in the duration of gestation or other outcomes such as proteinuric PIH, non-proteinuric PIH, or birth weight within the lowest 3% on the growth charts.	This study failed to support the hypothesis that fish oil supplementation improved pregnancy outcome in an at risk population for impaired fetal growth or PIH.
Bulstra Ramakers et al. 1995 [82]	To study the effects of adding 3 g/day of EPA to the diet, on recurrence rate of IUGR and PIH in a high-risk population	RCT, DB, PC.	Pregnant women with a history of IUGR with or without PIH in the previous pregnancy from the Netherlands *n* = 63	From 12 to 14 weeks of gestation until delivery	Intervention: 4 capsules 3 times daily, which corresponded to a daily dose of 3 g of EPA Placebo: Identical capsules with coconut oil	Compared to placebo, EPA supplementation resulted in (1) No difference in the rates of PTB	No information was provided about content of DHA No estimate of mean gestational length was provided
Olsen et al. 1992 [83]	To study the effect of a fish-oil supplement, a control olive-oil supplement, and no supplementation on pregnancy duration, birthweight, and birth length	RCT	Healthy pregnant women from Denmark *n* = 533	From gestation week 30 until delivery	Intervention: Four 1 g fish oil capsules/day containing 2.7 g n-3 fatty acids- 32% EPA, 23% DHA, 2 mg tocopherol Placebo: Four 1 g olive oil capsules/day No supplement group	Compared to placebo fish oil supplementation resulted in: (1) The highest mean length of gestation when all 3 groups were compared in a single analysis (fish oil, olive oil and no supplement: 283, 279.4 and 281.7 days respectively, *p* = 0.006). (2) On an average 4 days longer pregnancies in the fish-oil group compared to the olive oil group (95% CI: 1.5–6.4, *p* = 0.005). (3) The effect seemed to depend on the baseline intake of fish. -Among those 20% of the women who had the highest intake of fish at randomization, no difference could be detected between the oil groups. -In those 20% who had the lowest intake for fish, a difference of 7.4 days was observed (95% CI 2.2–12.6 days, *p* = 0.01). -In the middle 60%, the groups differed by 4.8 days (95 CI 1.8–7.8, *p* = 0.005).	Maternal baseline dietary intake could explain differences in the duration of gestation and higher intakes may have a saturating effect
Mardones et al. 2008 [84]	To study the effect of maternal food fortification with omega-3 fatty acids and multiple micronutrients on birth weight and gestation duration	Non-blinded, RCT, PC.	Healthy pregnant women up to 20 weeks Gestation from Chile *n* = 972	From up to 20 weeks of gestation until delivery	Intervention: 2 kg/month of powdered milk fortified with multiple micronutrients and both a-linolenic acid and linoleic acid; iron was supplied in an amino-chelated form Placebo: 2 kg/month powdered milk fortified with small amounts of iron sulphate, copper, zinc, and vitamin C.	Based on ITT analyses and in comparison with placebo, the intervention resulted in (1) Lower incidence of EPTB (0.4% vs. 2.1%; crude OR (95% CI): 5.26 (1.08–34.90), *p* = 0.02). (2) Increase in gestation duration (1.40 days difference, 95% CI: -0.02–2.82 d, *p* = 0.05). (3) Higher mean birth weight (65.4 g difference, 95% CI: 5–126 g; *p* = 0.03). (4) Higher infant length (0.37 cm difference, 95% CI: 0.06–0.68 cm, *p* = 0.019).	Impossibility to perform a blinded design and have strict control of compliance with the prescribed amounts of the products taken to the homes of the study subjects Slight difference in gestational age at recruitment Associations with gestation duration would need a larger sample size for confirmation (the statistical power reached only 0.61 in ITT analyses)
Smuts et al. 2003 [85]	To assess whether higher intake of DHA would increase duration of gestation and birth weight in US women	RCT, DB, PC.	Healthy pregnant women between the 24th and 28th week of pregnancy from the US (predominant black population) *n* = 291	From 24–28 weeks of gestation until delivery	Intervention: 1 DHA enriched egg/day (133 mg DHA) Placebo: 1 ordinary egg/day (33 mg DHA)	Compared to the placebo group, the supplementation with DHA-enriched egg resulted in (1) Increased duration of gestation (6.0 ± 2.3 days, *p* = 0.009) (based on analyses adjusted for maternal BMI at enrollment and number of prior pregnancies).	The unadjusted analysis showed a difference of 2.6 days (not statistically significant), while adjustment for maternal BMI at enrollment and number of prior pregnancies resulted in an increased duration of gestation by 6 days. The adjustments may have introduced a post hoc element into the interpretation of the result.

BMI: body mass index, DHA: docosahexaenoic acid, EPA: eicosapentanoic acid, EPTB: early PTB, PTB: preterm birth, IUGR: intrauterine growth retardation, LC-PUFA: long-chain polyunsaturated fatty acids, PIH: pregnancy-induced hypertension, RCT: randomized controlled trial, DB: double blind, PC: placebo controlled.

**Table 2:** Characteristics, efficacy and safety of clinical trials investigating the role of probiotics in reducing risk of any preterm delivery.

Ref.	Study				Ingredient, Daily Dose	Main Results	Comments
	Objective	Design	Population/Sample Size	Duration			
Gille et al. 2016 [145]	To assess whether probiotic supplementation with *Lactobacillus rhamnosus* GR-1 and *Lactobacillus reuteri* RC-14 can improve maternal vaginal microbiota	RCT, DB, PC	Healthy pregnant women (first trimester) from Germany, *n* = 320, 2010–2012	8 weeks to assess Nugent scores; entire pregnancy for PTB (secondary outcome)	Capsules with 10^9^ CFU, once daily	Compared to placebo, DHA supplementation resulted in No effect on vaginal microbiota (improvement in Nugent scores). No effect on PTB rates.	Low rate of preterm of 4% Very low rate of bacterial vaginosis 3%. Trend increase on miscarriages in treated (7.7% vs. 3.1%, *p *= 0.08).
Luoto et al. 2010 [146]	To assess whether dietary counselling and probiotic supplementation with (*Lactobacillus rhamnosus* GG and *Bifidobacterium lactis* Bb12) can improve pregnancy outcomes	RCT, PC 3 groups: (1) Probiotics and dietary counselling vs. (2) Placebo and dietary counselling (DB); (3) Placebo without dietary counselling (SB)	Healthy pregnant women in the first trimester from Finland, *n *= 256, late 1990s	From the first antenatal visit to the end of pregnancy	Capsules with 10^10^ CFU, once daily	Compared to placebo, probiotic supplementation resulted in 1. No effect on PTB rate.2. No effect on duration of gestation.	Very low rate of PTB: 1.7%.
Kraus Silva 2011 et al. [143]	To assess whether probiotic supplementation with (*L. rhamnosus* GR-1 and *L. reuteri* RC-14) can reduce BV and PTB	RCT, DB, PC	Pregnant women (8 to 20 weeks gestation), With asymptomatic BV: Vaginal pH >4.5, Nugent >4 from Brazil *n* = 644 randomized, late 1990s	<20 weeks gestation to 24 or 26 weeks	Capsules with 10^6^ colony-forming units each, twice daily	Compared to placebo, probiotics supplementation resulted in no effect on PTB rate. However, the PTB rates were lower with treatment (ITT: 1.6%, 5 in 304; vs. 3.3% 10 in 301)	Low rate of PTB 2.5% Low probiotics dose Exclusion criteria were very broad: previous history of PTB, hypertension, diabetes, asthma, cervical incompetence, atypical vaginal bleeding, atypical vaginal secretion, HPV, gonorrhea, syphilis, dysuria, pruritus, burning, corticotherapy, recent antibiotic therapy (within 8 weeks prior to screening)
Rautava et al. 2012 [148]	The effect of maternal administration of probiotics on atopic disease in infants.	RCT, DB, PC.	Pregnant women with atopic sensitization and either a history of or active allergic disease from Finland *n *= 241	Probiotics given to the mother 8 weeks before and 8 weeks after delivery.	(1) Dietary food supplement with *Lactobacillus rhamnosus* LPR + *Bifidobacterium longum* NCC 3001 (10^9^ CFU/day) (2) Dietary food supplement with *Lactobacillus paracasei *ST11 + NCC 3001 (1^9^ CFU/day) (3) Placebo	No information on preterm birth rates. Gestational age in all groups was 39 weeks with a similar range (34–41 weeks).	Not possible to draw firm conclusions about effects on preterm delivery. However, papers seems to suggest lack of effect because gestational ages were similar between groups.
Kim et al. 2010 [157]	The effect of maternal and infant administration of probiotics on atopic disease in infants	RCT, DB, PC.	Pregnant women with a family history of allergic diseases day *n *= 112, and their infants. from Korea	Probiotic was given to mothers from 8 weeks before delivery until 3 months post-delivery, then to infants from 4 months until 6 months	(1) Bifido Inc mix (*Bifidobacterium bifidum *BGN4, *Bifidobacterium lactis* AD011, *Lactobacillus acidophilus* AD030), 1.6 × 10^9^ CFU/day each, in powder (2) Placebo powder (maltodextrin and alpha-corn)	Infants delivered before 36 weeks were excluded. No difference observed in the number of infants removed between the two groups, suggesting no difference in PTB rates. In both groups the gestational ages were around 40 weeks, and birth weights were similar.	Not possible to draw conclusions about effects on PTB. However, papers seems to suggest lack of effect.
Ou et al. 2012 [158]	The effect of maternal administration of probiotics on atopic disease in infants	RCT, DB, PC	Pregnant women with atopic diseases history and Total IgE >100 kU/L from Taiwan *n *= 191	From 24 weeks gestation until delivery. After delivery, administration was exclusively to breastfeeding mothers	(1) *L. rhamnosus* GG (Valio, ATCC 53103) 10^10^ CFU/ day (2) Placebo (microcrystalline cellulose)	PTB rates were not reported. However, gestational age was 39 weeks in both groups (range 31–41 weeks in the *L. rhamnosus *GG group and 35–41 weeks in placebo group), which suggests lack of efficacy on PTB rates.	The study suggests that *L. rhamnosus *GG probably has no impact on PTB rates.
Vitali et al. 2012 [159]	The effect of probiotic supplementation during late pregnancy on vaginal microbiota and cytokine secretion	Non-randomized, controlled, pilot	Healthy pregnant women with no symptoms of vaginal or urinary tract infection from Italy *n *= 27	Probiotic was given during weeks 32–37 of gestation.	(1) Probiotic group: one sachet of VSL #3 (*Lactobacillus acidophilus, Lactobacillus plantarum, Lactobacillus casei, Lactobacillus delbrueckii ssp. bulgaricus, Bifidobacterium breve, Bifidobacterium longum, Bifidobacterium infantis, S. salivaris ssp. thermophilus*) (*n *= 12) 9 × 10^11^ total CFU/day (2) Control group: no supplementation (*n *= 12)	PTB rates were not reported, but the gestational ages were not different between the two groups. This suggests that the probiotic did had no effect on PTB rates. No significant changes were found in the amounts of the principal vaginal bacterial populations in women administered with VSL#3, but qPCR results suggested a potential role of the probiotic product in counteracting the decrease of *Bifidobacterium *and the increase of *Atopobium,* that occurred in control women during late pregnancy. Incidence of vaginal infections was not reported.	The study is too small to draw conclusions, but it did not show any effect of VSL3 on gestational age.
Stojanovic et al. 2012 [154]	The effect of probiotics on vaginal microflora, cervical length, cervical consistency, and fetal positioning.	Observational, randomized, prospective	Pregnant women	Probiotic was administered for 12 weeks during pregnancy	(1) untreated arm of the study (*n* = 30) (2) vaginal application of one tablet containing *L. rhamnosus* BMX 54 (Normogin™-(*n* = 30) once a week	No data on PTB rates as women were not followed until delivery. Increase in pathogenic microorganisms in the vaginal and/or cervical swabs of untreated women (*p *<0.05), also in average pH values (*p *<0.05), amount (*p *<0.05) and “whiff test” positivity (*p *<0.05) of vaginal discharge. Significant trend was also found for decrease in length (*p *<0.0001) and increase in dilatation (*p *<0.05) of cervix, as well as for lower position of the fetus (*p *<0.0001). In the group treated with *L. rhamnosus* BMX 54, none of these values significantly changed throughout the observation period, with the exception of cervical length that was significantly decreased at T3 (*p *<0.01).	Cannot conclude on PTB rates. However, it suggests that vaginally administered probiotic had a positive impact on parameters associated with PTB.

CFU: colony forming unit, RCT: randomized controlled trial, DB: double blind, PC: placebo controlled.

**Table 3:** Nutrients with known efficacy to reduce the risk of PTB.

Nutrient	Evidence for Efficacy	Dose	Duration	Comments
n−3 LC-PUFA (combinations of EPA and DHA)	26–61% reduction in the risk of early PTB	DHA: 133 to 2100 mg DHA/day EPA: 100 to 3000 mg EPA/day	Supplementation started between 12 to 30 weeks of gestation	Eight trials supplementing either DHA or EPA alone or using varying combinations of both (five trials in healthy pregnancies and three in at-risk pregnancies), two food-based interventions and 6 meta-analyses
DHA (predominantly DHA)	51.6% to 87.5% reduction in the risk of early PTB (<34 weeks)	600 to 800 mg DHA/day	Supplementation started <20 to 21 weeks of gestation	Two large RCTs available where PTB and EPTB were secondary outcomes and not the primary outcome.
Zinc	14% reduction in PTB	5 mg/day to 44 mg/day	Supplementations started from as early as before conception (one study) to at least starting before 26 weeks	Most studies were conducted in low income countries among women with poor nutritional status and likely to have low zinc concentrations. The reduction in PTB was not accompanied by reduction in LBW or a difference in the gestational age at birth.
Vitamin D	64% reduction in PTB	400 to 1000 IU/day (two trials), 60000–12000 IU (depending on baseline serum 25 (OH)D (one trial) cholecalciferol D3	Supplementation started between 20–30 weeks of gestation	The trials available were all of low quality.

DHA: docosahexaenoic acid, EPA: eicosapentanoic acid, EPTB: early PTB, PTB: preterm Birth, LBW: low birth weight.
